# Groove pancreatitis—a great mimicker

**DOI:** 10.1259/bjrcr.20150316

**Published:** 2016-05-07

**Authors:** Chakenahalli Nanjaraj, Biradar Basavaraj, Narayana Manupratap, Mysore Shashikumar, Narsipur Rajendrakumar, Mallaih PraveenKumar, Turamari Rashmi

**Affiliations:** Department of Radiodiagnosis, Mysore Medical College and Research Institute, Mysore, India

## Abstract

Groove pancreatitis is an uncommon form of chronic pancreatitis affecting the “groove” between the pancreatic head, duodenum and common bile duct. Many radiologists remain unfamiliar with this entity, with only a few descriptions of it existing in the radiology and pathology literature. The exact underlying cause of groove pancreatitis is unclear, although there are strong associations with peptic ulcer disease, smoking, long-term alcohol abuse, functional obstruction of the duct of Santorini and Brunner gland hyperplasia. This entity mimics pancreatic carcinoma and often ultimately leads to surgery. Hence it is important for radiologists to be familiar with imaging findings of groove pancreatitis to avoid diagnostic dilemma. Imaging findings in our case showed a soft tissue mass in the pancreaticoduodenal groove with enhancement, consistent with scar tissue and cystic changes within the lesion. It was associated with adjacent duodenal wall thickening with smooth and regular tapering of the pancreatic and common bile ducts.

## Summary

Groove pancreatitis is a form of chronic focal pancreatitis affecting the groove in the region of the pancreatic head, duodenum and common bile duct. The aetiology remains unclear, but it may relate to peptic ulcers, chronic alcohol abuse, smoking and gastric resection. Ever since its description, it remains a diagnostic dilemma for radiologists, pathologists and clinicians.

A definitive diagnosis remains challenging despite its imaging findings, and an inability to distinguish groove pancreatitis from a primary duodenal, ampullary or pancreatic malignancy often ultimately leads to exploratory laparotomy. CT scan and MRI play a vital role in recognizing this entity, which presents as plate-like scar tissue in the groove region accompanied by duodenal wall thickening on the pancreatic side and cysts within the lesion, which may simulate groove pancreatic carcinoma. The lack of infiltration/encasement of peripancreatic vessels^1^ with smooth and regular tapering of the pancreatic and common bile ducts helps in distinguishing groove pancreatitis from carcinoma.

## Case report

**Figure 1.**
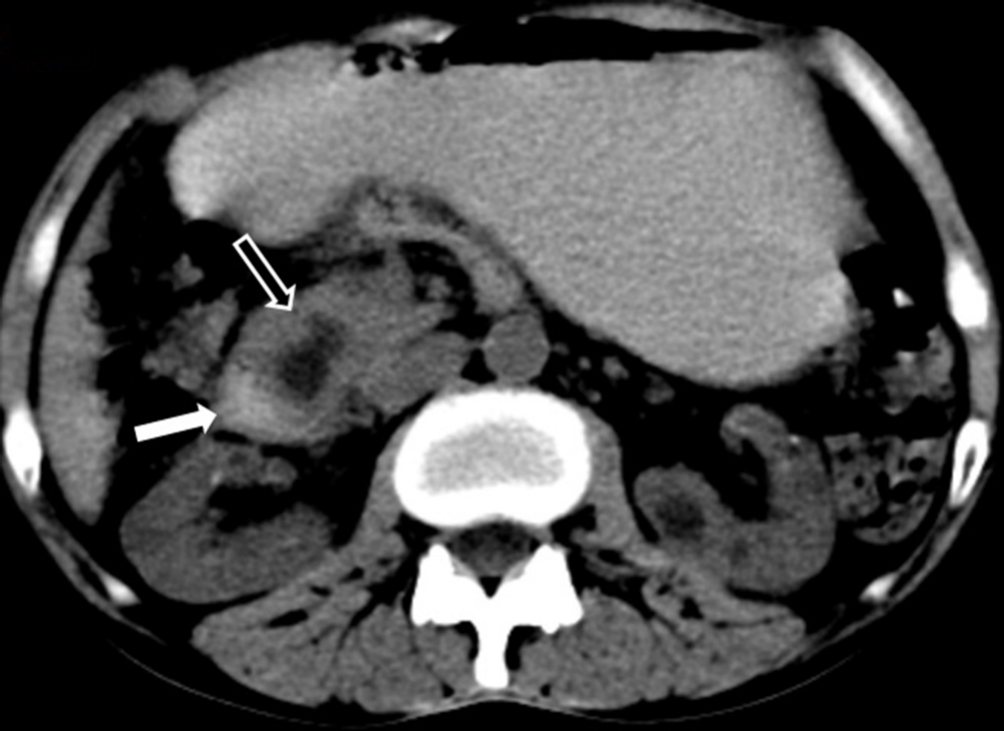
Axial unenhanced CT scan showing soft tissue density lesion (black arrow with white outline) with a cystic lesion within the lesion in the pancreaticoduodenal groove with poorly defined fat planes with adjacent duodenum (white arrow) and pancreas.

**Figure 2.**
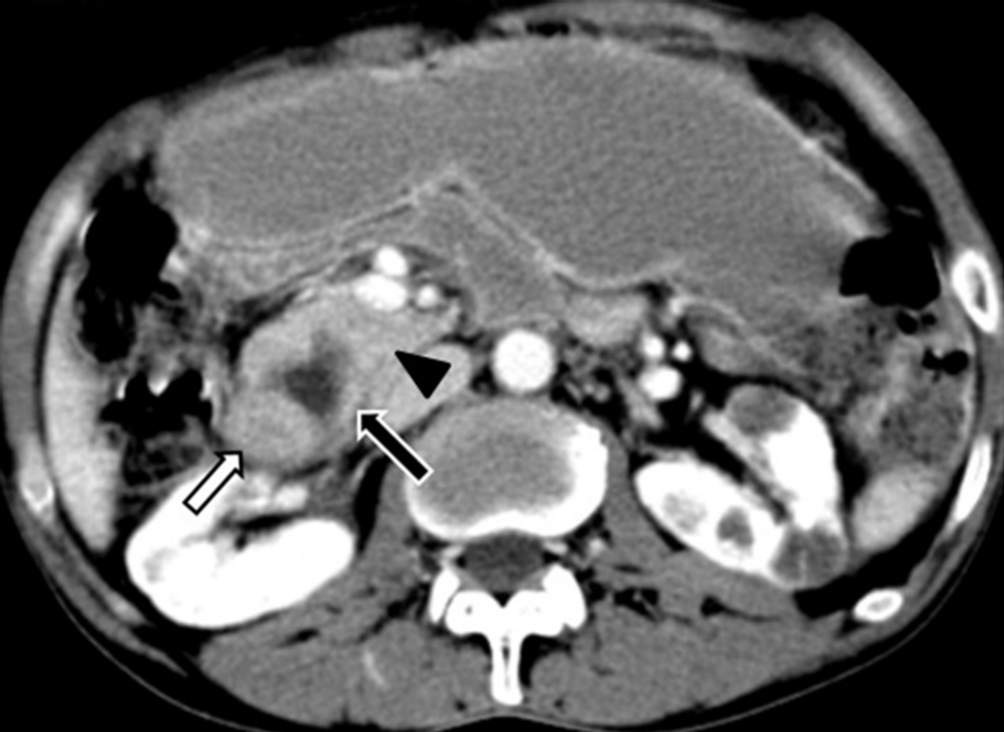
Axial contrast-enhanced CT image (portal venous phase) showing a minimally enhancing lesion with cystic change within (black arrow with white outline) the pancreaticoduodenal groove with adjacent duodenum showing wall thickening (white arrow with black outline) and poorly defined fat plane with pancreatic head and uncinate process (black arrowhead). Incidentally, few simple cortical cysts were also noted in both kidneys.

**Figure 3.**
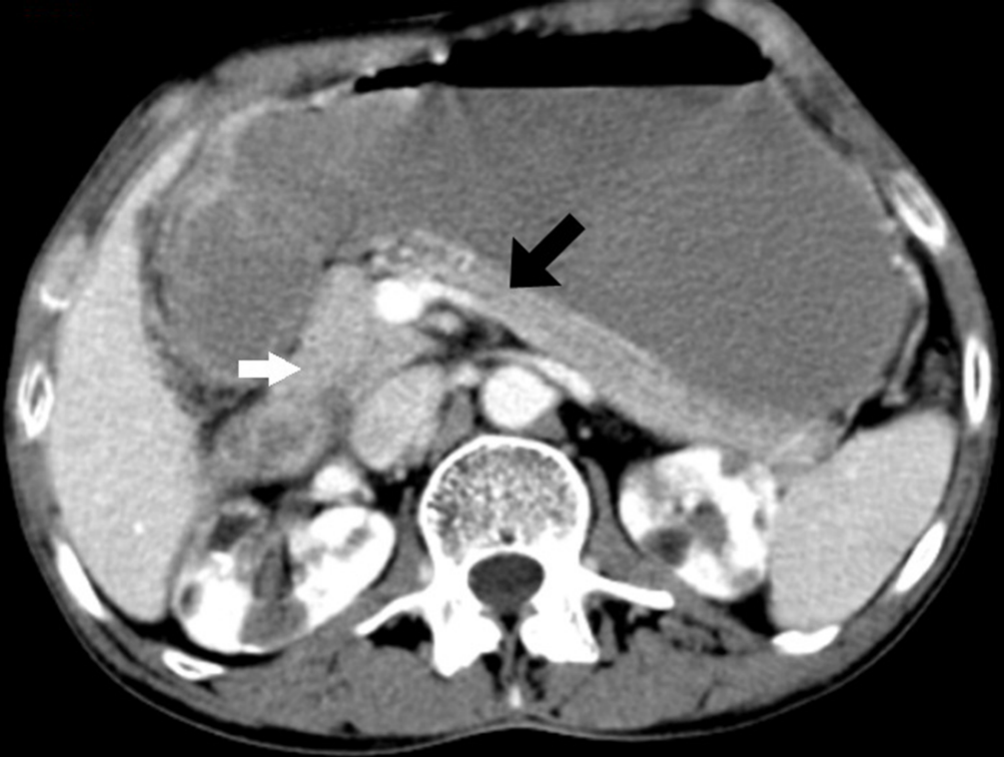
Axial contrast-enhanced CT image (portal venous phase) showing a soft tissue density lesion (white arrow) in the pancreaticoduodenal groove that merges imperceptibly with the head of the pancreas, but the rest of the pancreatic body and tail (black arrow) shows normal appearance with no evidence of duct dilatation.

**Figure 4.**
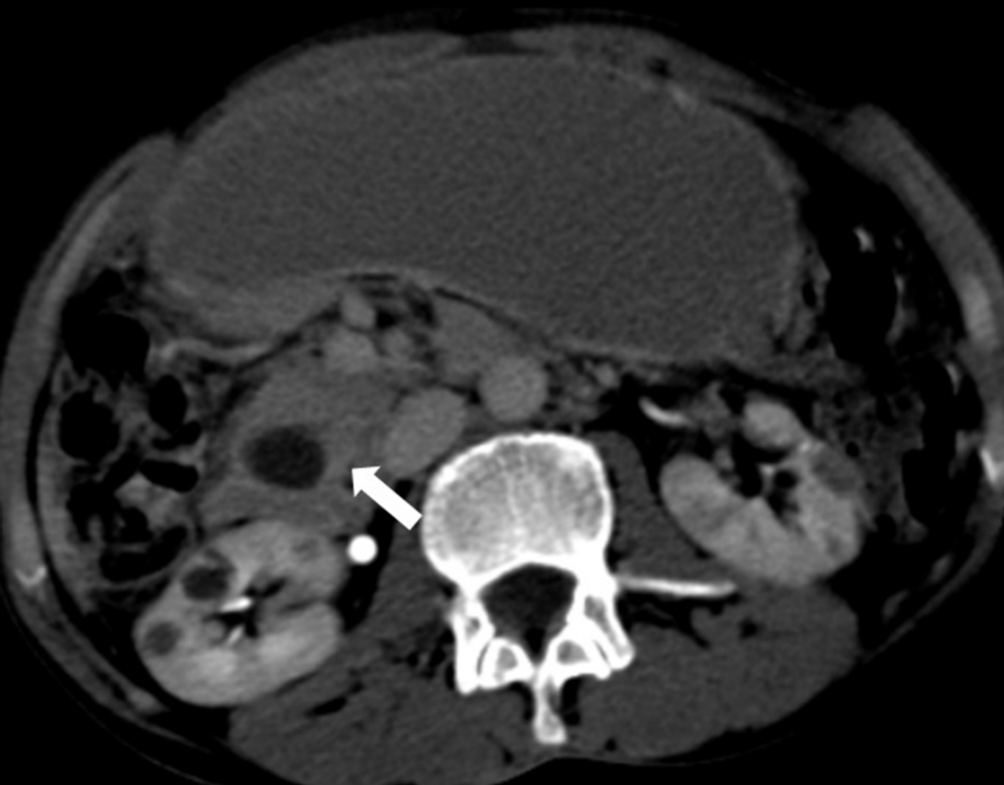
Axial contrast-enhanced CT image showing persistent mild enhancement of the lesion consistent with fibrous scar tissue in delayed phase imaging (arrow).

**Figure 5.**
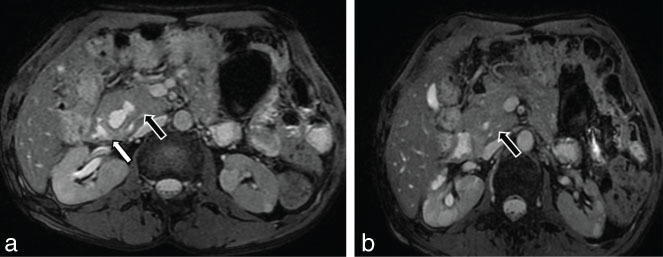
(a, b) Axial two-dimensional fast imaging employing steady-state acquisition fat-saturated images showing a soft tissue intensity lesion with cystic change within in the pancreaticoduodenal groove that merges imperceptibly with the head of the pancreas (black arrow with white outline) and poorly defined fat plane with adjacent duodenum (white arrow).

**Figure 6.**
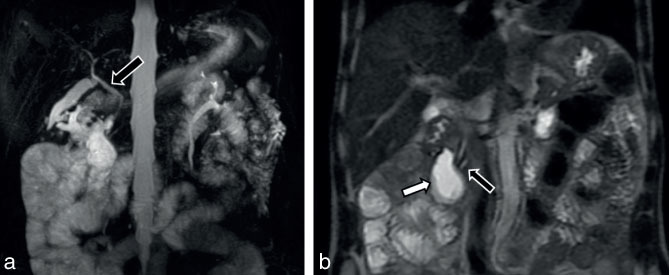
(a) Coronal reformatted MR cholangiopancreatographic image showing normal common bile duct calibre (black arrow with white outline). (b) Coronal reformatted MR cholangiopancreatographic image showing a cystic lesion within the soft tissue intensity lesion (white arrow with black outline) and adjacent smooth and regular tapering of the pancreatic and common bile ducts (black arrow).

A 50-year-old male presented with complaints of chronic, intermittent abdominal pain. The patient had a 20-year history of mild-to-moderate amount of alcohol consumption. There was no history of jaundice, fever or weight loss. His laboratory tests revealed no significant abnormal findings. Serum amylase and lipase, and tumour markers [CA 19-9, carcinoembryonic antigen (CEA)] were within normal limits. The patient was subjected to a CT examination. On unenhanced CT image, a poorly defined soft tissue mass was seen in the pancreaticoduodenal (PD) groove with a hypodense cystic lesion within the mass (Figure 1). On post-contrast study, the lesion showed minimal enhancement in the portal venous phase (Figures 2 and 3), but delayed imaging at 2–3 min showed mild persistent enhancement of the lesion compared with the pancreatic parenchyma that was consistent with scar tissue (Figure 4) and a non-enhancing cystic lesion within the lesion. The lesion showed poorly defined fat planes with the adjacent second part of the duodenum on its right side and pancreatic head on the left side. Mild thickening of the wall of the second part of the duodenum adjacent to the lesion with variable luminal narrowing was noted. The common bile and pancreatic ducts appeared grossly normal. The pancreatic body and tail were normal. In order to clearly delineate the ductal system and the periampullary region, and to know the extension of the lesion, the patient was also subjected to an MRI examination. Axial two-dimensional fast imaging employing steady-state acquisition (Figure 5a,b) sequences showed soft tissue signal intensity lesion in the PD groove with a cystic lesion within the lesion and the aforementioned CT scan findings. MR cholangiopancreatography sequence (Figure 6a,b) revealed smooth and regular tapering of the pancreatic and common bile ducts. The gallbladder was distended and the cystic duct was normal. Incidentally, a few simple cysts (Bosniak 1) were noted in both the kidneys. The patient was advised further surgical intervention but he refused and was managed with conservative treatment. At present, the patient is asymptomatic.

## Discussion

Groove pancreatitis was first described by Becker in 1973.^2^ Groove pancreatitis is a form of chronic focal pancreatitis affecting the PD groove. The PD groove is a potential space between the head of the pancreas, the duodenum and the common bile duct.^3,4^ Groove pancreatitis is categorized into two forms: pure and segmental. The pure form of groove pancreatitis affects only the groove, whereas the segmental form involves the head of the pancreas, with scar tissue located in the groove.^4–6^

Groove pancreatitis usually affects males in the fourth or the fifth decade of life and its pathogenesis remains unclear, although several factors such as chronic alcohol abuse, smoking, peptic ulcers, gastric resection, true duodenal-wall cysts and pancreatic heterotopia in the duodenal wall may be related to this condition.^4,5^ Our patient also had a 20-year history of chronic alcohol use. Disturbed outflow through the duct of Santorini owing to chronic alcohol toxicity^7^/alcohol-induced periampullary Brunner gland hyperplasia causing occlusion or dysfunction of the minor papilla/pancreatic heterotopia is usually regarded as a causative factor.^5,6,8^

Clinically, patients present with chronic intermittent abdominal pain similar to chronic pancreatitis; a few of them may present with recurrent vomiting, which is attributed to variable luminal narrowing owing to duodenal wall thickening and impaired motility. Overt jaundice is uncommon in groove pancreatitis unlike pancreatic carcinoma, which presents with progressive jaundice. The pancreatic enzymes tend to remain normal or marginally elevated.^9,10^ In our case, the pancreatic enzymes were within normal limits.

Histopathological analysis of groove pancreatitis reveals the presence of scar tissue with fibrosis in the PD groove.^7^ Cystic lesions, either true cysts or pseudocysts, are frequently encountered in the groove or the duodenal wall, with hyperplasia of the Brunner glands, dense myxoid stromal proliferation resembling pancreatic hamartoma, myoadenomatosis, and inflammation and fibrosis of the upper and posterior part of the pancreatic head. The duodenum is always involved by a chronic inflammatory process, with scar tissue in the wall leading to fibrosis and variable levels of luminal narrowing. The origin of cystic changes is controversial but cystic dystrophy of heterotopic pancreas in the duodenal wall remains the most accepted theory.^8,10,11^

In 2004, the term “paraduodenal pancreatitis” was suggested by Adsay and Zamboni^11^ to unify the same lesional spectrum or different stages of the same disease that primarily affected the duodenum in the region of minor papilla, such as cystic dystrophy of the duodenum, paraduodenal cysts, groove pancreatitis and pancreatic hamartoma of the duodenum.

CT scan, MRI and endoscopic sonography are modalities commonly used in the assessment of pancreatic masses. A contrast-enhanced CT scan shows soft tissue attenuation material between the duodenum and the pancreatic head, which shows delayed enhancement. Additional CT scan features include thickening of the duodenal wall and, occasionally, paraduodenal cysts. In the segmental form, there can be a hypodense non-enhancing focal lesion within the upper part of the pancreatic head that is difficult to differentiate from pancreatic carcinoma.^5,12^ MRI findings include a sheet-like hypointense mass on *T*_1_ weighted images that is isointense or slightly hyperintense relative to the pancreatic parenchyma on *T*_2_ weighted images.^3,10,13^

Irie et al^3^ described the MRI features of five patients with groove pancreatitis with the aforementioned MRI findings, and histological analysis revealed that these imaging features correlated with fibrous scar in each of the five patients.

On MR cholangiopancreatography, the common bile and pancreatic ducts show a normal calibre with regular and smooth tapering. An interesting finding reported by Blasbalg et al^10^ is a banana-shaped gallbladder that appears elongated and distended; as groove pancreatitis is not usually associated with a significant degree of biliary dilatation, distention of the gallbladder is usual.^5^ On endoscopic sonography, groove pancreatitis has a varied appearance, including a hypoechoic or heterogeneous mass along the descending duodenum with periduodenal and intramural fluid collections.^14,15^

Several differential diagnoses need to be borne in mind when assessing a pathology of the PD groove that presents with cystic changes. These include duodenal diverticula, duodenal duplication cyst, enlarged necrotic node and pancreatic pseudocyst. Knowledge of the features of each disease is very essential, as it allows one to arrive at a specific diagnosis that helps in deciding the clinical management and prevent unnecessary surgical intervention.^16^ It is important to interpret the imaging findings very cautiously because small periampullary carcinomas/pancreatic carcinoma may mimic groove pancreatitis. At times, the hypoechoic mass imperceptibly merges with the head of the pancreas and it becomes difficult to differentiate groove pancreatitis (especially the segmental form) from pancreatic carcinoma, particularly in those cases of pancreatic carcinoma that have a significant fibrous component and show delayed enhancement. The presence of smooth, progressive narrowing of the main pancreatic duct as it courses through the head of the pancreas can help differentiate groove pancreatitis from a pancreatic malignancy, which causes abrupt irregular duct narrowing with pronounced dilatation of the upstream duct; also, clinically, pancreatic carcinoma usually presents with progressive jaundice and elevated tumour markers.^17^ Other differential diagnoses include duodenal cancer and cholangiocarcinoma of the distal common bile duct.

Treatment options are categorized into conservative therapy and surgical intervention.^5,18,19^ Conservative therapy is primarily useful during the early stages or acute phase of the disease and includes abstinence from alcohol and smoking, use of analgesics, proton pump inhibitors^1^ and a pancreatic enzyme supplement, nutritional support and endoscopic cyst drainage or stenting. Surgical intervention is generally reserved for those with progressive and non-relieving obstructive gastrointestinal symptoms or doubtful cases where exclusion of malignancy is difficult. Preferred surgical intervention includes pancreatoduodenectomy, which not only relieves gastrointestinal obstruction but also chronic abdominal pain. Casetti et al^18^ reported complete remission of abdominal pain in 76% of patients following pancreatoduodenectomy.

## Conclusions

Groove pancreatitis is an uncommon form of chronic focal pancreatitis affecting the “groove” between the pancreatic head, duodenum and common bile duct. MRI and contrast-enhanced CT scan remain the imaging modalities of choice in the study of the PD groove. The intention of this case report is to make this entity and potential anatomical space more familiar to radiologists and clinicians, which would aid in making a correct pre-operative imaging diagnosis and reduce further diagnostic work-up such as invasive biopsy, thereby limiting the overall patient risk, delayed diagnosis and apprehension. Combining the characteristic imaging appearances with clinical information plays a crucial role in making the diagnosis and developing a treatment plan.

## Learning points

1. Groove pancreatitis is a benign, uncommon form of chronic focal pancreatitis that affects the potential anatomic space called the PD groove. Laboratory findings reveal no significant abnormality or marginal elevation of the pancreatic enzymes.
2. In a patient with a history of chronic alcohol abuse, the presence of a soft tissue density lesion in the groove with cystic change within the lesion and adjacent duodenal wall thickening is highly suggestive of groove pancreatitis.
3. The presence of regular tapering of the pancreatic and common bile ducts in groove pancreatitis in contrast to the abrupt, irregular ductal stenosis or complete ductal obstruction seen in pancreatic carcinoma plays a key role in differentiating it from the latter.

## References

[1] Zaheer A, Haider M, Kawamoto S, Hruban RH, Fishman EK Dual-phase CT findings of groove pancreatitis. Eur J Radiol 2014; 83: 1337–43. 24935140 10.1016/j.ejrad.2014.05.019 PMC4316673

[2] Becker V, Bauchspeichel D Spezielle Pathologische Anatomie Bd. VI Doerr W, Seifert G, Uhlinger E, eds. Berlin, Germany: Springer; 1973 252–445.

[3] Irie H, Honda H, Kuroiwa T, Hanada K, Yoshimitsu K, Tajima T, et al MRI of groove pancreatitis. J Comput Assist Tomogr 1998; 22: 651–5. 9676462 10.1097/00004728-199807000-00027

[4] Stolte M, Weiss W, Volkholz H, Rösch W A special form of segmental pancreatitis: "groove pancreatitis". Hepatogastroenterology 1982; 29: 198–208. 7173808

[5] Arora A, Rajesh S, Mukund A, Patidar Y, Thapar S, Arora A, et al Clinicoradiological appraisal of ′paraduodenal pancreatitis′: pancreatitis outside the pancreas!. Indian J Radiol Imaging 2015; 25: 303–14. 26288527 10.4103/0971-3026.161467 PMC4531457

[6] Becker V, Mischke U Groove pancreatitis. Int J Pancreatol. 1991; 10: 173–82. 1787332 10.1007/BF02924155

[7] DeSouza K, Nodit L Groove pancreatitis: a brief review of a diagnostic challenge. Arch Pathol Lab Med 2015; 139: 417–21. 25724040 10.5858/arpa.2013-0597-RS

[8] Chatelain D, Vibert E, Yzet T, Geslin G, Bartoli E, Manaouil D, et al Groove pancreatitis and pancreatic heterotopia in the minor duodenal papilla. Pancreas 2005; 30: e92–e95. 15841034 10.1097/01.mpa.0000161885.79373.1d

[9] Shudo R, Yazaki Y, Sakurai S, Uenishi H, Yamada H, Sugawara K, et al Groove pancreatitis: report of a case and review of the clinical and radiologic features of groove pancreatitis reported in japan. Intern Med 2002; 41: 537–42. 12132521 10.2169/internalmedicine.41.537

[10] Blasbalg R, Baroni RH, Costa DN, Machado MCC MRI features of groove pancreatitis. AJR Am J Roentgenol 2007; 189: 73–80. 17579155 10.2214/AJR.06.1244

[11] Adsay NV, Zamboni G Paraduodenal pancreatitis: a clinico-pathologically distinct entity unifying "cystic dystrophy of heterotopic pancreas", "para-duodenal wall cyst", and "groove pancreatitis". Semin Diagn Pathol 2004; 21: 247–54. 16273943 10.1053/j.semdp.2005.07.005

[12] Itoh S, Yamakawa K, Shimamoto K, Endo T, Ishigaki T CT findings in groove pancreatitis. J Comput Assist Tomogr 1994; 18: 911–15. 7962798 10.1097/00004728-199411000-00011

[13] Castell-Monsalve FJ, Sousa-Martin JM, Carranza-Carranza A Groove pancreatitis: MRI and pathologic findings. Abdom Imaging 2008; 33: 342–8. 17624569 10.1007/s00261-007-9245-x

[14] Wronski M, Karkocha D, Slodkowski M, Cebulski W, Krasnodebski IW Sonographic findings in groove pancreatitis. J Ultrasound Med 2011; 30: 111–5. 21193712 10.7863/jum.2011.30.1.111

[15] Tio TL, Luiken GJ, Tytgat GN Endosonography of groove pancreatitis. Surg Laparosc Endosc 1991; 23: 291–3. 10.1055/s-2007-1010691 1743134

[16] Yu J, Fulcher AS, Turner MA, Halvorsen RA Normal anatomy and disease processes of the pancreatoduodenal groove: imaging features. AJR Am J Roentgenol 2004; 183: 839–46. 15333380 10.2214/ajr.183.3.1830839

[17] Raman SP, Salaria SN, Hruban RH, Fishman EK Groove pancreatitis: spectrum of imaging findings and radiology-pathology correlation. AJR Am J Roentgenol 2013; 201: W29–W39. 23789694 10.2214/AJR.12.9956 PMC4005339

[18] Casetti L, Bassi C, Salvia R, Butturini G, Graziani R, Falconi M, et al “Paraduodenal” pancreatitis: results of surgery on 58 consecutives patients from a single institution. World J Surg 2009; 33: 2664–9. 19809849 10.1007/s00268-009-0238-5

[19] Arvanitakis M, Rigaux J, Toussaint E, Eisendrath P, Bali MA, Matos C, et al Endotherapy for paraduodenal pancreatitis: a large retrospective case series. Endoscopy 2014; 46: 580–7. 24839187 10.1055/s-0034-1365719

